# EUS-FNA Diagnosis of a Metastatic Adult Granulosa Cell Tumor in the Stomach

**DOI:** 10.1093/labmed/lmac024

**Published:** 2022-04-08

**Authors:** Ilias P Nikas, Athanasia Sepsa, Evangelia Kleidaradaki, Charitini Salla

**Affiliations:** School of Medicine, European University Cyprus, Nicosia, Cyprus; Department of Pathology, Metropolitan Hospital, Athens, Greece; Cytopathology Private Practice, Thessaloniki, Greece; Department of Cytopathology, Hygeia and Mitera Hospital, Athens, Greece

**Keywords:** cytopathology, ovarian cancer, immunohistochemistry, metastasis, cytology, neoplasm

## Abstract

Granulosa cell tumors are uncommon ovarian neoplasms, predominantly of the adult type (AGCT). In this report, we present a rare case of a patient with metastatic AGCT to the stomach diagnosed with endoscopic ultrasound–guided fine-needle aspiration (EUS-FNA). A 61-year-old woman without a history of AGCT underwent both a vaginal and an abdominal ultrasound that showed a solid and cystic ovarian mass along with a solid mass in the gastric antral wall. Subsequently, an EUS-FNA was performed to assess the gastric lesion. Cytologic findings showed high cellularity, and the groups of neoplastic cells invaded the muscle layer of the stomach. Notably, these cells formed Call-Exner bodies, whereas some nuclei exhibited nuclear grooves. Immunohistochemistry was performed, revealing positivity for α-inhibin, calretinin, and CD56 in the neoplastic cells, whereas chromogranin, synaptophysin, CD117, and DOG1 were negative. The combination of clinical presentation, radiology, cytomorphology, and immunohistochemistry could facilitate the diagnosis of metastatic AGCT and the management of such patients.

Granulosa cell tumors are uncommon ovarian neoplasms, comprising approximately 2% to 5% of all ovarian cancers. They belong to the family of sex cord stromal tumors and are predominantly adult granulosa cell tumors (AGCT; 95%), rather than the juvenile type (5%). Studies have shown that AGCT present clinically with symptoms and signs caused by the presence of an adnexal mass, including abdominal pain or swelling.^1-3^ Because AGCT are hormonally active, they secrete high levels of estrogen, often resulting in abnormal vaginal bleeding, and they pose a higher risk of patients developing endometrial hyperplasia and cancer.^3,4^ They are considered low-grade and indolent ovarian malignancies that grow slowly, and staging is their most important prognostic factor. Most patients are diagnosed at stage I, exhibiting a favorable prognosis.^1-3^ Of interest, 5- and 10-year survival rates of patients with AGCT have been reported to be 98% and 84%, respectively. Therefore, these patients exhibit a much better prognosis than patients with other more common ovarian cancers, such as serous ovarian carcinomas.^5^ However, patients with AGCT require a long-term follow-up because this neoplasm may behave unpredictably and exhibit aggressive behavior in the long term. Notably, AGCT can recur or metastasize even many years after their initial detection; this may happen in patients initially diagnosed at stage I as well. Metastases of AGCT are mostly confined to the area of the pelvis and abdominal cavity (eg, peritoneum or omentum), yet more distant sites have also been reported, such as the liver, lung, and bones.^1,2,6^

Fine-needle aspiration (FNA) is a modality that has been successfully utilized in the diagnosis of metastatic AGCT.^2,7-11^ In this report, we present a case of a patient with metastatic AGCT to the stomach diagnosed with endoscopic ultrasound–guided (EUS) FNA. This is the first cytomorphologic description in the literature of a metastasis to this site diagnosed with this procedure.

## Case Description

A 61-year-old woman without a history of AGCT underwent both a vaginal and an abdominal ultrasound, which showed a solid and cystic ovarian mass and a solid mass in the gastric antral wall, respectively. Subsequently, an EUS-FNA was performed to assess the gastric lesion.

**Figure 1.**
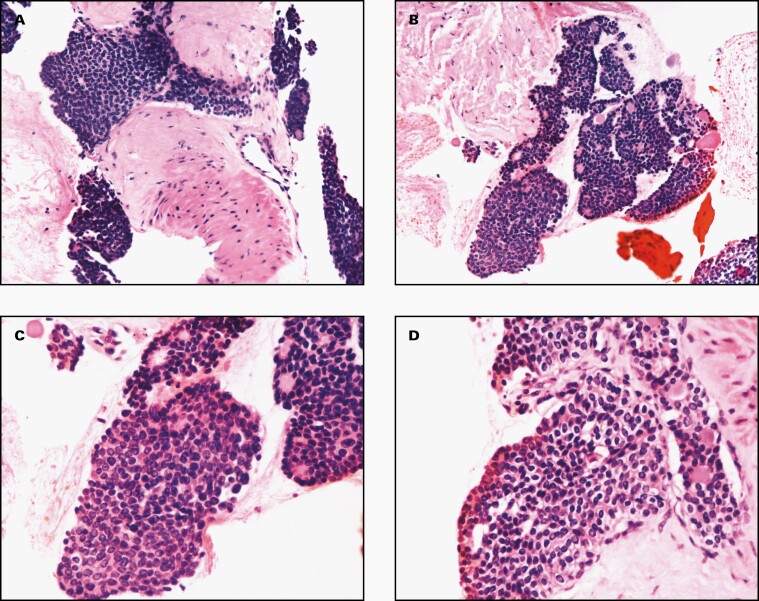
The H&E cell block cytomorphology of a metastatic adult granulosa cell tumor in the stomach wall. The neoplastic cells were mostly arranged in syncytial groups invading the muscle layer of the stomach and also exhibited a tendency to form Call-Exner bodies. A few of the neoplastic cells exhibited nuclear grooves (A and B, ×200; C and D, ×400).

The material received was solely used to prepare a cell block. Subsequent H&E-stained slides showed high cellularity. The neoplastic cells were mostly arranged in syncytial groups invading the muscle layer of the stomach and also exhibited a tendency to form rosette-like structures (**Figure 1**). Neoplastic nuclei showed a monotonous appearance with ovoid shape and minimal atypia, and some of them exhibited nuclear grooves. Cytoplasm was of moderate amount, and the cell borders were ill-defined. No necrosis was found. The combination of clinical history (presence of a solid and cystic ovarian mass) and cytomorphology raised the possibility of an AGCT metastasis to the stomach. In this situation, the rosette-like structures would represent Call-Exner bodies. In the latter, the neoplastic granulosa cells are arranged around small lumens containing eosinophilic material, as we saw in our patient (**Figure 1**).

**Figure 2.**
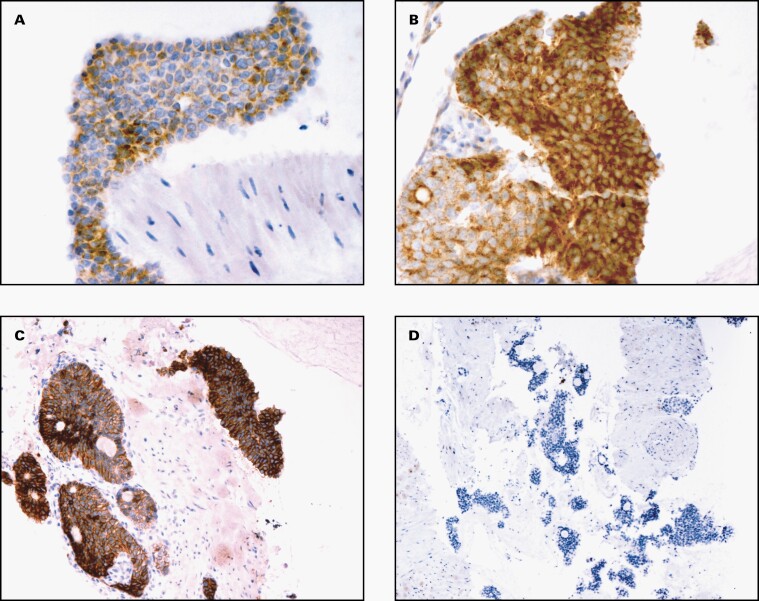
A selection of immunohistochemical stains performed on the cell block material of a metastatic adult granulosa cell tumor in the stomach wall (A, α-inhibin ×400; B, calretinin ×400; C, CD56 × 200; D, chromogranin ×100).

Immunohistochemistry was performed on the cell-block material. The neoplastic cells were positive for α-inhibin, calretinin, and CD56 (**Figure 2**). In contrast, they were negative for chromogranin, synaptophysin, CD117, and DOG1.

Therefore, by combining the radiologic, cytomorphologic, and immunohistochemical findings, we developed a diagnosis of a metastatic AGCT to the stomach.

## Discussion

Research has shown that AGCT are low-grade ovarian malignancies derived from the granulosa cells of the ovarian follicles. Although most exhibit an indolent behavior, long-term follow-up is required because a few can recur or metastasize even many years after the initial diagnosis.^1,2,6^ Clinical history may be unavailable to the pathologist; thus, a diagnosis of metastatic AGCT can be difficult to make, especially when the specimen cellularity is inadequate for ancillary studies.^2,8^ A few case series and reports describing cytologic diagnoses of metastatic AGCT have been published, where the latter has been found in sites such as the liver, lungs, bone, omentum, bowel, bladder, spleen, kidney, pleural and ascitic fluids, and lymph nodes.^2,7-11^ A recent case series has effectively summarized the published literature on metastatic AGCT diagnosed with cytology.^2^

A diagnosis of metastatic AGCT can be suspected when the cytomorphology is classic, including the presence of Call-Exner bodies and nuclei with grooves.^2^ Immunochemistry can confirm this suspicion because AGCT cells will most likely be positive for α-inhibin, calretinin, and CD56.^12^ Notably, the detection of the *FOXL2* mutation (missense point mutation; 402C→G), by either immunohistochemistry or sequencing, is an accurate diagnostic biomarker and pathognomonic for AGCT. Furthermore, *FOXL2* immunohistochemistry is more sensitive than α-inhibin and calretinin, besides being highly specific to highlight the presence of AGCT.^1,13,14^

For our patient, we formed our differential diagnosis list based on the location of the mass inside the stomach wall and the low-grade cytomorphology of the neoplasm. Low-grade lesions growing in the submucosa/muscularis can include gastric neuroendocrine tumors (NET), gastrointestinal stromal tumors (GIST), leiomyomas, and schwannomas. Studies have shown that NET are composed of cells with “salt and pepper” nuclei, albeit without grooves, that are positive for chromogranin and synaptophysin with immunohistochemistry.^15^ Whereas GIST are positive for DOG1 and CD117, leiomyomas and schwannomas exhibit spindle-shaped morphology and immunopositivity for desmin and S100, respectively.^16^

## Conclusion

In conclusion, AGCT are malignant ovarian neoplasms with indolent behavior, yet they have an unpredictable malignant potential that prompts their long-term follow-up. The combination of clinical presentation, radiology, cytomorphology, and immunohistochemistry can facilitate the diagnosis of metastatic AGCT and the management of such patients.

## Abbreviations:

AGCT: adult granulosa cell tumors
EUS-FNA: endoscopic ultrasound–guided fine-needle aspiration
NET: neuroendocrine tumors
GIST: gastrointestinal stromal tumors
